# Semen extenders: An evaluative overview of preservative mechanisms of semen and semen extenders

**DOI:** 10.14202/vetworld.2021.1220-1233

**Published:** 2021-05-20

**Authors:** Ghadeer Sabah Bustani, Falah Hasan Baiee

**Affiliations:** 1 College of Dentistry, The Islamic University, Najaf, Iraq; 2 Department of Clinical Science, Faculty of Veterinary Medicine, University of Kufa, Kufa 54003, Najaf, Iraq

**Keywords:** additives to semen extenders, artificial insemination, cryopreservation, egg yolk-based extenders, lipid peroxidation, semen extenders

## Abstract

Reproduction is fundamental for all living things as it ensures the continued existence of a species and an improved economy in animal husbandry. Reproduction has developed since history, and diverse processes, such as artificial insemination and in vitro fertilization, have been developed. Semen extenders were discovered and developed to protect sperm from harmful factors, such as freeze and osmotic shock, oxidative stress, and cell injury by ice crystals. Semen extenders preserve sperm by stabilizing its properties, including sperm morphology, motility, and viability and membrane, acrosomal, and DNA integrity. Therefore, semen extenders must provide a favorable pH, adenosine triphosphate, anti-cooling and anti-freeze shock, and antioxidant activity to improve semen quality for fertilization. Hence, this review provides precise data on different semen extenders, preservative mechanisms, and essential additives for semen extenders in different animals.

## Introduction

Artificial insemination (AI) is a powerful and unique technique for fertilizing the females of most mammals. The first animal was conceived using AI in 1784, and the first trial to produce straw for AI was at the beginning of the 20^th^ century [1]. AI was intended to increase the number of insemination doses from a single ejaculate but was unsuccessful because of the absence of sperm-washing procedures for both chilled or frozen–thawed semen of the male donor. These techniques are considered the foundation stone in the history of AI [1]. Moreover, AI is widely applied in cattle [2] and humans [3] but less in sheep [4], buffalos [5], horses [6], deer [7], and other mammals [8]. To improve economic efficiency, AI can impact different productivity projects, such as cattle and sheep husbandry and increasing weaning weight. Therefore, AI has a remarkable ability to impact economic feasibility [9].

Furthermore, AI needs fresh or well-preserved semen, and 95% of all AI is accomplished using preserved semen [10]. Thus, semen must be preserved in a perfect medium to maintain its quality [10-12]. Accordingly, it is necessary to develop and evaluate semen extenders used to preserve semen during chilling or cryopreservation [13].

This review provides precise data on semen extenders, semen preservation mechanisms, and essential additives for semen extenders in different animals.

## Preservation of Semen

The two primary techniques for semen storage are chilling and cryopreservation. For the chilling technique, semen is stored at 4-5°C for 3 days for maximum and best results. In the cryopreservation technique, semen is exposed to freezing for 3 h at 4°C. Meanwhile, it is filled into 0.25-mL straws and finally preserved and stored in liquid nitrogen for years [14]. Therefore, the crucial factors for long-term semen preservation to retain its quality include cooling for 2-3 h, adding a cryoprotectant, and freezing in liquid nitrogen [15].

## Cooling Temperature for Semen Preservation

Temperature is decreased to induce sperm inactivity during storage in liquid form. The globally accepted cooling temperature is between 4°C and 5°C to maintain semen quality and reduce gamete metabolism in dogs[16], stallions [17], bulls [18], goats [19], and rams [20], whereas in boars, the diluted liquid semen must be kept between 15°C and 17°C [21]. Mammalian sperm cells, especially for rams [22], boars [23], bulls [24], stallions [12], and men [25], are damaged by rapid temperature drop. The temperature of post-chilled semen may not be as ­harmful to the intactness of the male sperm as the post-thawed technique, which results in low spermatozoal value [26]. A crucial advantage linked to using chilled semen is the high fertility percentage compared with the freeze-thawed semen, which decreases the insemination dose and increases the dose number, therefore, reducing storage expenses and easing the use of AI [27]. Moreover, the lifespan of chilled semen in the female reproductive tract is longer than that of frozen semen and with a higher fertilization rate [28]. Singh *et al*. [29] found that soybean-based diluent at 25% gave good quality values for bull semen at 5°C at different time intervals. Studies showed that the supplementation of different soybean lecithinconcentrations in Tris-based semen extenders (containing 2% virgin coconut oil) increased the functional parameters of post- chilled bull semen [30].

## Cryopreservation of Semen

Cryopreservation is the freezing of sperm, a technique used to keep cells and tissues in a vital state at −196°C in liquid nitrogen. The use of liquid nitrogen started in the modern cryobiology era [31]. Furthermore, other advantages of cryopreservation include the long-distance transportation of valuable genetic materials and preventing the spread of pathogens [32].

Cryopreserved bull semen has been used commercially in cattle for decades since the data illustrated that the conception rate of the cryopreserved semen using AI technique is acceptable compared with ­natural mating [33,34]. Thus, a single dose of cryopreserved semen can achieve an equivalent *in vivo* fertilization 8 times more than that of fresh semen [35,36]. Semen cryopreservation generates free radicals due toexposure to atmospheric oxygen, and removes seminal plasma from the sperm cells. This results in the production of lipid peroxidation from sperm cells and, consequently, increases in reactive oxygen species (ROS) formation [37]. Furthermore, semen cryopreservation has no standard protocol applied in most previous studies [38,39].

The improvement in semen extenders and cryopreservation techniques has significantly reduced the harmful effects of cryopreservation. However, cryopreservation still causes sperm damage in humans and various animals [40,41].

## Factors that Determine Sperm Cryo-survival

The challenge during cryopreservation is not sperm cells’ ability to resist the storage period in liquid nitrogen but crossing the intermediate temperature zone between −15°C and −60°C. The cells must go through these temperatures twice, first during cooling and then thawing, causing injurious effects on the integrity of the sperm plasma membrane, acrosome, and nucleus; mitochondrial function; and sperm motility [32,42].

### Cryopreservation techniques

Different cryopreservation techniques include conventional freezing, directional freezing, and sperm vitrification [43].

#### Conventional freezing techniques

Calling star-shaped ice crystal freezing is used in the manual freezing technique [43], where the semen is placed in contact with liquid nitrogen vapor at a height 4-5 cm above for ≤15-10 min before storage in liquid nitrogen. The freezing rate is approximately 60°C/min [15,43,44]. The conventional technique varies among species depending on sperm quality or cryo-survivability after freeze–thawing [44].

#### Directional freezing techniques

This technique is based on a multi-thermal gradient approach [45,46]. After dilution with a freezing extender, semen is chilled to 4-5°C at 0.3°C/min and packaged in prechilled hollow tubes (5, 8, or 12 mL). Tubes are advanced through a linear temperature gradient, from 5°C to −50°C at a constant velocity of 1 mm/s. Then, seeding is done for 60 s through a cold block at its other end, and tubes are moved to a collection chamber (−100°C). These tubes are collected and transferred into liquid nitrogen for storage [43,47].

#### Sperm vitrification techniques

Vitrification is a process of transforming a solution containing a high concentration of cryoprotectant in a glass-like state without ice crystal formation through an ultra-rapid cooling process [48]. The high cooling rate, high viscosity, and low volume enable vitrification for sperm preservation [49]. In humans, vitrified sperm exhibits high post-thaw quality. Hence, vitrification is introduced by solidifying the solution into a glassy state without causing any crystallization in a fast and inexpensive manner. Holding solution is a significant component for vitrification prepared with N-2-hydroxyethylpiperazine-N-2-ethane sulfonic acid-buffered Medium 199 and 20% of calf bull serum [50].

## Cryoprotectants Substance

The various anti-freezing cryoprotectants are dimethyl sulfoxide (DMSO) and propylene glycol. During freezing and thawing of isolated cells, mostly 5-15% cryoprotectant concentration must be maintained for better results [51].

### Development of semen extender

Experts have developed media for sperm survival during the cooling and freezing techniques [15]. Many challenges, such as media toxicity [52], irregular pH [53], ROS [54], energy source [55], sperm membrane damage [24], and cryo-shock preservatives [56], were faced and have been resolved. Extenders protect sperm, conserve motility, and fertility over time by stabilizing the plasmalemma, provide energy substrates, and prevent harmful effects of pH and osmolarity changes [57]. These solutions facilitate the increase in fertilization rate using high-quality extenders during chilling and cryopreservation. Therefore, a better quality of semen extenders and additives should be added to increase semen quality and increase the rate of sperm fertilization [15,58].

### Semen extender

Semen extenders are used as a medium for preserving sperm to enable fertilization. Semen extenders can also maintain and preserve sperm metabolic processes, control the pH of the medium during and after post-thawing, control bacterial transmission and contamination, and reduce cryogenic damage [10,59]. Likewise, semen extenders must provide other characteristics, such as maintaining the pH at 6.8-7.2 [53], provide energy [60], antioxidant to reduce the oxidative stress [61], antibiotics to prevent contamination [8], and anti-freezing shock [62,63]. These properties keep the sperm storage and transport and enable it to be used in AI *in vitro* fertilization, intracytoplasmic sperm injection, and research studies. Extenders appear in two forms: Chilled (liquid form) for an average of 3 days and cryopreserved for years [64]. At present, several extenders use different material sources such as animal source, egg yolk, skimmed milk [65], and plant source (soybean lecithin) [24], which provide various features and diverse problems according to the type of sperm extender and species. A good trait of soybean lecithin semen extender compared with the egg yolk extender is its more hygienic nature [66]. Egg yolk semen extenders are extensively used in the laboratory and field techniques because of their reasonable price and satisfactory results [24].

## Various Components of Semen Extender

### Non-penetrating cryoprotectant source

Skim milk and egg yolk are widely used as non-penetrating cryoprotectants for preserving sperm [67] of different male mammals, such as rams [68], bulls [59], stallions [65,69], boars [70], and humans [29]. Because these extenders might protect sperm membranes, their acrosome and DNA may be damaged because of high lecithin content [71]. Furthermore, soybean lecithin extenders can substitute the animal source as a source of lipid/lipoprotein [72].

### Egg Yolk

The first professor who used egg yolk in semen extender was Philips in 1939 [73,74]. Egg yolk is the primary non-penetrate substance used in extenders to dilute semen and protect sperm from freeze shock during the chilling process. Egg yolk-based extender is commonly used in chilled, frozen semen, or both. It works as a reservoir of cholesterol and phospholipids that help protect the sperm cell membrane and acrosome against cryogenic injury. Furthermore, it prevents the loss of membrane phospholipids during the freezing process [24,75]. Egg yolk protein has hydrophobic properties, which cannot penetrate the cell wall of sperm. The low-density lipoprotein (LDL) of egg yolk maintains sperm membrane phospholipids throughout the cryopreservation processes [24,76]. The previous works have shown that sperm are protected during freezing by sequestering lipid-binding proteins from LDL in the egg yolk [10,77,78]. It is also considered a source of long-chain polyunsaturated fatty acids [79]. Besides, egg yolk contains lipid, protein and carbohydrate; it also contains minerals [10].

In contrast, several drawbacks against egg yolk-based semen extender use include the wide range of variability in the composition of egg yolk, the risk of disease transmission or bacterial contamination, and involvement of egg yolk in the microscopic examination of semen [39]. In general, egg yolk is used in semen extenders at different concentrations. However, it was also used at 20% (v/v). Pieces of evidence revealed that LDLs are the egg yolk active ingredients responsible for sperm protection [39,62].

Two types of extenders can be prepared from egg yolk:

### Egg yolk-based extender

Here, the concentration of the egg yolk is 20% [24,62,80]. Tris-buffered egg yolk extenders containing fructose and glycerol preserve the fertility of animal sperm at high extension rates. Poultry egg yolk can be used in the semen extenders; however, some studies show the possibility to use different egg yolks of animals, such as quail [81], turkey [82], duck [83], pigeon [82], goose [84], and ostrich [85]. Furthermore, researchers found that quail’s egg yolk was improved the quality of semen higher than other birds egg yolk in terms of sperm motility and membrane integrity in ram [86] and bull [82] semen. Nonetheless, Swelum *et al*. [87] found that chicken egg yolk extenders are most recommended for buck semen.

### LDL extender

LDL (w/v) was prepared in the laboratory following the method described by Moussa *et al*. [88]. They used poultry egg yolks to isolate LDL by ultracentrifugation [22,88]. Higher kinetic parameters were achieved using 2%, 4%, and 8% LDL compared with 20% whole egg yolk in a Tris-milk extender and can lower the concentration of LDL, such as 2% associated with skimmed milk, which can be used for buffalo semen freezing [89].

According to several studies, egg yolk can be used with a 20% extender concentration [24,82,90]. In contrast, LDL can be used with 8% concentration [91,92], and 2% LDL can be added solely with skimmed milk [10,89]. One significant challenge of using egg yolk and its derivatives is microbial contamination by *Escherichia coli*. Consequently, the fertilization capacity of contaminated semen could negatively affect the risk of microbial contamination associated with the egg yolk extender [93].

## Milk Sources

Milk has been adapted for freezing mammalian semen mostly in a reconstituted form combined with arabinose, fructose, or egg yolk [10,44]. Skim milk proteins buffer semen pH and may also chelate any heavy metal ions [94]. An important milk compound is lactose, which is hydrophilic [95] and cannot diffuse the cell wall of the sperm cells, which protects the cell wall and prevents freeze shock [94-96]. Skim milk-based extender is superior to Tris-based extender based on semen preservation [96].

## Soybean Lecithin

Soybean lecithin is an alternative for egg yolk and has been developed and used commercially for semen preservation [97]. Lecithin from soybean has been successfully used for semen cryopreservation [98]. Nowadays, researchers believe that extenders free from animal ingredients can decrease the risk of contamination transported by the animal source [93,99]. Therefore, soybean lecithin can be used as an alternative to milk- or egg yolk-based extenders for semen cryopreservation in bulls [24,30,98], boars [100], rams [101], dogs [102], deer [103], and camels [104].

The active components of soybean lecithin and egg yolk are entirely identical. These components are oleic acid, palmitic acid, stearic acid, and phosphatidylcholine. They prevent the diluted semen from freeze shock. The prevailing phospholipids in most mammalian biological membranes can confer physical stability to sperm cells [105]. On the basis of the research results of Gamal *et al*. [97] and Miguel-Jimenez *et al*. [98], soybean lecithin-containing diluter is possibly the best semen extender in bulls. Furthermore, El-Keraby *et al.* [106] found that the use of soybean-based extender increases sperm functional motility and reduces bacterial contamination in freeze–thawed bull semen. In a similar vein, Zhang *et al*. [107] ensured that the semen extender supplemented with soybean lecithin at 6% could upgrade sperm general and progressive motility and intact plasma membrane of post-thawed male boar sperm cells. Conversely, Singh *et al*. [26] and Rehman [71] discovered that 25% soybean-based extender could improve sperm motility, sperm viability, sperm membrane integrity, and acrosomal integrity of bull sperm at 5°C.

Depending on the results of Fathi *et al*. [108], using Tris-soybean lecithin-based extender at a 3% concentration can be an appropriate alternative to either BullXcell^®^ or OptiXcell^®^ in Damascus goat sperm cryopreservation.

## Glycerol as an Anti-shock

After discovering glycerol as a remarkable cryoprotective agent for cryopreserving semen, using liquid nitrogen for adequate storage of frozen semen and AI has been a valuable and prevalent reproductive biotechnology for cattle genetic improvement [24]. Discovering the cryoprotective properties of glycerol in 1949 enabled the cryopreservation of different animals’ sperm. [109]. Although different cryoprotective substances have been tested, including DMSO and propanediol, glycerol remains the favorite cryoprotectant for semen cryopreservation. Glycerol is a dominant cryoprotectant that can cross the cell membrane [101]. Studies stated that glycerol could be added to the semen at different temperatures. For instance, Evans *et al*. [110] observed that good semen protection was achieved at 30°C. In contrast, another suggested that procedure for semen cryopreservation is adding glycerol at 5°C [14,111]. Furthermore, studies also suggested that freezing extenders containing 3% glycerol combined with the straw freezing method using dry ice can produce the best post-thaw quality parameters for boar semen [112].

Fernández *e*t *a*l. [113] revealed that semen extenders containing egg yolk with 6% glycerol, followed by a rapid cooling rate, could yield higher post-thaw outcomes for epididymal sperm compared with semen extenders containing 3% glycerol. In another study, Martínez *et al*. [114] demonstrated that extenders containing 4% glycerol with 10% egg yolk are most suitable.

DMSO is a cryoprotectant that quickly enters sperm cells. It can be used to maintain the frozen sperm quality of bulls [115], boars [29], goats [116], and dogs [117]. In stallions, it is preferable to use DMSO or methyl formamide alone or combined with glycerol in skim milk extenders as cryoprotectants and alternatives to extenders containing glycerol only [118,119]. A study that compared three cryoprotectants, glycerol, ethylene glycol, and DMSO, revealed that the post-thaw motility and fertilization capacity of glycerol were higher than those of ethylene glycol and DMSO in dogs [120]. Earlier studies revealed that ethylene glycol is more dominantly used in buffalos [87], bulls [121], and sheep [122].

## Source of Energy

Energy intake is responsible for the continuation of development and the function of all living cells, and gametes are no exception. Two metabolic pathways are producing adenosine triphosphate (ATP), that supplies energy for the main functions of sperm, which are oxidative phosphorylation and glycolysis [123]. Glycolysis occurs in the cytoplasm of sperm cells and provides energy for sperm metabolism [123]. Sugar, such as fructose and glucose, is considered the primary energy source in sperm cells [124]. However, fructose is the best sugar for maintaining functional membrane integrity, adequate sperm motility, andtonic after thawing [125].

Disaccharides are considered non-permeating agents for cells. These sugars interact with phospholipids of the plasma membrane, increasing sperm survival post-cryopreservation [126]. Moreover, lactate and pyruvate are significant energy sources in stallion sperm with dose effects on mitochondrial function, motility, and ROS production [127]. Trehalose can be used as a cryoprotectant in semen extenders to preserve the optimal quality of motility, viability, and membrane integrity of goat sperm cells compared with other types of sugar [128]. Glucose is a component of egg yolk and, therefore, can be used as an energy source. Nonetheless, other kinds of sugars, such as galactose, sucrose, maltose, xylose, and raffinose, have been successfully used for frozen bull semen [10,11].

## Various Components as Additives to Semen Extenders

To promote the quality of the extenders, several studies have been undertaken to use different materials and compounds such as those of plant origins [24,129-131], whole milk [54], fish oil [132], and honey [133], which contain natural compounds such as antioxidants [24,130,131].

### Added Components of Plant Origin

There is an international demand for using natural medical sources in semen extenders of different animals [134,135], for example, strawberry [136], green tea [134], virgin coconut oil [30], pomegranate [135], and *Pinus brutia* [137,138], among others. The effects of several plant extracts on fertility have been demonstrated as antioxidants in many animal species due to their free radical scavenging properties [139].

According to the results of El-Sheshtawy [136], he used a 1%–5% concentration of strawberry in Tris extender and improved semen parameters in cooling temperature and used strawberry at 3-6% concentration, which improved semen parameters in freezing temperature for bull semen.

Researchers found that supplementation of Tris-citric acid extender with 1.0% green tea improved sperm parameters in both *in vitro* and *in vivo* fertilization, which decreased lipid peroxidation in buffalo bull sperm freezing and thawing processes [134].

For virgin oil addition, researchers found that Tris-based extenders containing 2% virgin oil did not improve the quality of parameters for freeze–thawed bull semen but enhanced the quality of parameters for chilled bull semen [30]

An experimental study illustrated that adding *P*. *brutia* to the semen extender does not improve parameters such as motility but prevents chromatin damage and reduces oxidative stress and sperm abnormalities when used at a concentration of 50 mg/mL [137].

### Added components of animal origin

#### Honey

Malik [133] found that adding honey to extenders significantly affected sperm motility before freezing and sperm abnormality of the freeze–thawed semen. Honey contains a high number of various simple sugars and antioxidants [140]. Furthermore, honey is also a highly concentrated product. It has a potential hyperosmotic extracellular environment around sperm cells that enhance the efflux of intracellular fluid, thereby minimizing the formation of ice crystals inside the sperm cytoplasm, which has been linked to sperm damage during cryopreservation [141]. This illustrated that using honey increased the quality of the semen after thawingcompared with using egg yolk extenders.

Yimer *et al*. [142] showed that adding honey to bull semen Tris extender at 2.5% was optimum to obtain improved semen cryopreservation results compared with Bioxcell^®^. Another experiment [143] illustrated that adding different honey concentrations to extenders has various effects on the semen quality of different bull breeds. Jersey bull exhibited the best sperm quality compared with Mafriwal, Piedmontese, and Limousin bulls. Because El-Nattat et al. [144] considered that 1% concentration of honey additive to Bioxcell^®^ extender could be higher effectively when used in bull semen cryopreservation compared with Bioxcell^®^ only identified as a control.

Moreover, Fakhrildin and Alsaadi [141] showed that adding of honey to the freezing semen medium of humans at 10% concentration to semen extender resulted in enhanced sperm post-thawing quality of most sperm parameters.

In addition, researchers have reported the benefits of using honey as a supplement in the cryopreservation semen media of various animals, such as goat [145], which act as natural antibiotics against pathogenic bacteria, hinder sperm survival, fertilizing ability, reduce the number of dead abnormal sperm, and acrosomal damage. Some studies showed that using 2.5% honey might be an energy source to ram semen [146]. El-Sheshtawy *et al*. [147] illustrated that adding 3% of honey in extenders as a cryoprotectant improved Arab stallion post-thawing sperm parameters.

#### Fish oil

Fish oil can improve semen performance after freeze–thawing and AI besides the type of extender shown in bulls. The addition of 150 mg/100 mL fish oil in the extender could positively enhance the quality of post-thawed semen of Kalang swamp buffalos [59]. Abdi-Benemar *et al*. [148] found that adding 0.30 g of fish oil per 100 mL of egg yolk-based extender resulted in an improved fertility capacity of ram and goat semen. Kaeoket *et al*. [149] found that adding fish oil to boar semen extender improves sperm motility, viability, and acrosomal integrity. The researcher also showed that fish oil supplementation has a beneficial effect on semen quality, except for *in vitro* evaluations [150].

Some studies reported that the addition of fish oil to feed supplements improved the semen quality and fertility rate of sheep [151], bulls [152], rams [153], boars [154], and stallions [155].

#### Vitamins

Vitamins are added to extenders to improve semen function parameters for liquid nitrogen storage or cryopreserved sperm cells because vitamins are non-enzymatic antioxidants [156-159].

#### Vitamin B_12_

Adding Vitamin B_12_ to the extenders improved bull frozen semen quality, elevated the motility percentage of sperm cells, and improved movement characteristics [68,86,160,161]. Researchers found that the addition of 2.50 mg/mL Vitamin B to semen extenders improved bull frozen semen parameters and quality [161]. Furthermore, oral administration of 200 mg/kg body weight per day of Vitamin B_12_ could improve fresh and post-thawed sperm quality and fertility in male broiler breeders [159].

#### Vitamin E

Vitamin E is a cellular stabilizer of unsaturated lipids against oxidative deterioration, and hence, it maintains the structural and functional integrity at the subcellular level [161,162]. In general, Vitamin E is the primary component of the antioxidant system in sperm cells [163,164]. Furthermore, adding Vitamin E to Tris-egg yolk extenders at 60 and 120 µM provides higher integrity to the plasma membrane, mitochondria, and kinematic parameters of sperm cells of rams [165], roosters [162], and bulls [164] post-cryopreservation.

#### Vitamin C

Vitamin C is the most crucial antioxidant in seminal fluid [166]. Researchers found that 0.9 mg/mL of Vitamin C improves the longevity and quality of chilled sperm in Awassi ram semen stored at 5°C. Furthermore, as an alternative to glutathione, Vitamin C is considered more efficient in protecting ram sperm viability and acrosomal integrity than Vitamin E because Vitamin c can neutralize H_2_O_2_ production in a hydrophilic environment [167,168] by preventing peroxide formation [169]. However, higher concentrations of Vitamin C (2.5 mM) proved to be harmful to sperm motility in freeze–thawed bull semen [168]. Studies in humans showed that a high concentration of Vitamin C in the range of 0.02-0.6 mM adversely affected sperm motility [170].

### Other Additions

Several researchers have added various substances to semen extenders, such as milk, caseinate, and lactoferrin [120]. Other researchers added hormones, such as insulin [171], follicle-stimulating hormone [172], and testosterone [173], to the semen extenders. The addition of selenium improved male reproductive performance by potentiating semen quality and suppressing free radicals [174,175]. Selenium could decrease lipid peroxidation and increase antioxidants in rooster seminal plasma after the freeze–thawing process [176].

#### Antibiotic addition to semen extenders

Antibiotics are added to semen extenders to reduce microbial contamination of the external environment or during semen collection. Different antibiotics, such as penicillin and streptomycin, ceftiofur, apramycin, and aminoglycosides or linco-spectin + tylosin + gentamycin, have been added to semen extenders [10].

#### Commercial extenders

Several commercial extenders are used for diluting and preserving semen during cooling and cryoprotection. Optidyl^®^ and Triladyl^®^ (Biovet, France) are commercial extenders containing egg yolk and provide excellent protection for bull semen against freeze shock. It is usually used by many French AI centers [76]. Bioxcell^®^ is a commercial extender that contains milk, egg yolk, or both [177]. Gent^®^ A (Minitüb GmbH, Tiefenbach, Germany) is a commercial extender containing egg yolk and is used for the preservation of semen for a long time [178]. BotuSemen^®^ is a commercial extender that contains a skim milk base used for preserving frozen sperm of stallions [12]. EquiPlus^®^ is a commercial extender that contains defined milk proteins used to preserve semen [26]. INRA 96^®^ is a commercial extender that contains a caseinate used to preserve semen [179].

## Semen Assessment

### Sperm motility

The motility of sperm is the most critical parameter for evaluating male potential fertility [77], and it depends on mitochondrial function [180,181]. Motility includes total general motility, progressive motility, and kinematic parameters [181]. Sperm cells must possess high maintenance of sperm motility to ensure maximum potential fertility [114,182]. Moreover, the motility of sperm cells is also associated with sperm DNA defects. Furthermore, sperm motility is crucial for fertilization [45,181]. Immotile sperm and motility disorders of sperm are notable indicators of male infertility [183]. Therefore, microscopic examination and estimation of the percentage of forward movement of sperm are a standard test to indicate male fertility [45,181].

The tail of mammalian sperm cells is represented by a single, specific type of motile cilium known as the sperm tail (flagellum) that generates its movement to propel the cell through the female reproductive tract and fertilize the oocyte [184]. Sperm cells rely on vigorous motility that is initiated once they are released. They reach capacitation, which is needed to hyperactivate the ability to perform the acrosome reaction and fertilization [161,162]. In general, the tail motion is generated by ATP. When activated, sperm cells exhibit vigorous motility and enter rapid consumption of intracellular energy in which ATP content could be relevant for fertilizing potential [185].

Computer-assisted sperm analysis has been used to collect data after reducing human inequality during semen estimation. Moreover, it could be noted that different sperm motilities deem a fundamental part of semen evaluation in almost all creatures [186]. The most critical kinetic parameters include curve–linear velocity, straight-line velocity, and average-path velocity [182].

### Sperm viability

The viability of sperm cells is an essential aspect of ejaculation quality that determines competitive fertilization success, proportional to live sperm [187]. Spermatozoal viability is essential for motility and fertilizing ability. Once sperm viability is reduced, their ability to induce fertilization is decreased [58,187]. The percentage of live sperm cells was determined in the laboratory by identifying the number of sperms that did not take up the eosin–nigrosin stain [14,58,187].

### Sperm morphology

Sperm morphology depends on spermiogenesis [188] or events that occur after spermiation [175]. Improper handling of semen samples because of lack of experience can cause challenges during the cooling and freezing processes. Furthermore, these challenges may lead to acrosomal damage and abnormality of sperm tails [189]. Sperm shape abnormality includes head defects, such as microcephaly and macrocephaly. Midpiece defects, such as proximal cytoplasmic droplets, distal midpiece reflexes, and segmental aplasia of the mitochondrial sheath, are common [190].

There are three theories about abnormal sperm that can be presumptive. The first theory suggests primary or secondary defects; the primary defect occurs at the seminiferous tubules in the progress of spermatogenesis. In contrast, the secondary sperm abnormality is caused by abnormal function of the epididymis or during semen handling after ejaculation [139]. The second theory ascribes the sperm defect to the relationship between sperm and the fertility of males. Therefore, it is defined as a major or minor defect. The major sperm defect can directly affect male fertility; however, the minor defect may not affect male fertility [191]. Whether the defects of sperm cells are compensable or un-compensable, it distinguishes between the types of sperm defect. This may be compensated for by increasing the numbers of sperm to overcome sperm defects [192]. The third theory states that sperm defects can be classified according to the defect site (head, midpiece, and tail). Sperm morphology can be evaluated either with non-stained wet models under phase-contrast microscopy orfixed and stained sperm cells with post-dried eosin–nigrosine stain under 100× magnification [193].

## Acrosomal Reaction

The study of acrosomal integrity in mammalian species is a valuable tool in evaluating male subfertility and infertility. With advancements in microscopic visualization and cell-staining technology, methods for determining acrosomal integrity have been developed. The acrosome reaction is an exocytotic event initiated when sperm binds to the zona pellucida of an ovum. Once sperm binding occurs, the outer acrosomal membrane fuses with the overlying plasma membrane of the ovum [194]. The fusion of these membranes triggers vesiculation, a process where many small vesicles are created that allows for the dispersal of acrosomal enzymes [195,196]. The release of acrosomal enzymes enables the sperm cell to digest its way through the zona pellucida and begin the process of fertilization [197,198].

## Importance of an Intact Acrosome

The ability of a sperm cell to undergo capacitation, acrosome reaction, and a fertilization event requires an intact acrosome at the time of ejaculation and after the freeze-thawing process. The disruption or damage to the acrosome is permanent and results in premature loss of acrosomal contents, ultimately preventing fertilization [199,200]. Moreover, damaged acrosomes do not undergo vesiculation properly but spontaneously rupture and defect fertilization in the end [199].

## Hypo-osmotic Swelling Test (HOST)

The HOST evaluates the functional integrity of the sperm’s plasma membranes. Although the HOST is a simple test, it is considered an indicator of fertility in some species as the viability of the sperm membrane is an essential requirement for fertilization [50,201]. HOST and the integrity of the acrosome could be associated with motility results. To evaluate the sperm membrane integrity using HOST, an aqueous solution is prepared with fructose and trisodium citrate in distilled water to produce a solution (100 mOsm/kg H_2_0) as described by Lamia *et al*. [202] and Kumar *et al*. [99].

## *In vivo* Fertility Evaluation

It is crucial to examine the *in vivo* fertility test to provide comprehensive and perfect data. This test is considered the most important one for evaluating semen quality and provides information on the capability of sperm and pollination capacity of the ova after access to the female reproductive tract. Potential fertility depends on multiple parameters that require a multi-parametric analysis of sperm morphology, sperm motility, membrane status of sperm, sperm acrosome reaction, and genome integrity of sperm to provide a complete picture of a male’s fertility potential. Moreover, the reliability of fertility prediction is reported to increase by combining several *in vitro* sperm quality parameters [180,181,203]. The difficulty of this assay is that it is a time-consuming and costly procedure as hundreds of successful inseminations are required.

## Lipid Peroxidation Test

A typical trait of biological cell membranes is the asymmetrical organization of fatty acids (lipids) within the bilayer. The composition of lipids in most mammalian sperm cell membranes is different compared with that in somatic cells. Polyunsaturated fatty acids are found in high amounts in sperm cell membranes, and the ratio of saturated to unsaturated fatty acids in ruminants’ sperm cell membranes is higher than in other animals. This makes sperm cell membranes more subject to damage by peroxidation, primarily when an ROS is present [37,204]. In contrast, controlling the release of molecular oxygen may result in less ROS production and hence maintain the fertilizing ability, acrosome reaction, and capacitation of sperm [37,205].

## Conclusion

Our review article provides a comprehensive analysis of different semen extenders, the mechanisms of preserving semen, and essential additives for semen extenders in different animals. The study can be used as a road map for future studies to develop appropriate semen preservation and AI techniques.

## Authors’ Contributions

FHB: Conceptualized, drafted, and supervised the final version as well as editing of the review. GSB: Collected relevant literature, contributed to the original draft, data curation, investigation, and review of the manuscript. All authors read and approved the final manuscript.
